# Radiological Assessment After Pancreaticoduodenectomy for a Precision Approach to Managing Complications: A Narrative Review

**DOI:** 10.3390/jpm15060220

**Published:** 2025-05-28

**Authors:** Fabrizio Urraro, Vittorio Patanè, Alfredo Clemente, Nicoletta Giordano, Damiano Caputo, Roberto Cammarata, Gianluca Costa, Alfonso Reginelli

**Affiliations:** 1 Department of Life Sciences, Health and Health Professions, Link Campus University, 00165 Rome, Italy; f.urraro@unilink.it (F.U.); a.clemente@unilink.it (A.C.); 2 Department of Precision Medicine, University of Campania “L. Vanvitelli”, 80138 Naples, Italy; patavittorio@gmail.com (V.P.); nicoletta.giordano@unicampania.it (N.G.); alfonso.reginelli@unicampania.it (A.R.); 3 Department of Medicine and Surgery and Research Unit of General Surgery, Università Campus Bio-Medico, 00128 Rome, Italy; d.caputo@policlinicocampus.it (D.C.); r.cammarata@policlinicocampus.it (R.C.); 4 Operative Research Unit of General Surgery, Fondazione Policlinico Universitario Campus Bio-Medico, 00128 Roma, Italy

**Keywords:** biliary leakage, computed tomography, delayed gastric emptying, magnetic resonance cholangiopancreatography, magnetic resonance imaging, pancreatic fistula, pancreatic surgery, pancreaticoduodenectomy, postoperative complications, radiological assessment

## Abstract

Radiological assessment following pancreaticoduodenectomy is critical for the prompt diagnosis and management of postoperative complications, significantly influencing patient outcomes. Pancreaticoduodenectomy, or the Whipple procedure, is the standard surgical intervention for pancreatic and periampullary malignancies, but it involves notable risks, especially from complications like fistulas, bleeding, or leakage. Cross-sectional imaging, particularly contrast-enhanced computed tomography, serves as the primary diagnostic tool due to its rapid acquisition, high resolution, and effective delineation of postoperative anatomy and complications. Magnetic resonance imaging (with cholangiopancreatography and hepatobiliary contrast agents) complements CT by providing superior contrast resolution for specific complications, notably in the biliary system and pancreatic duct. This narrative review discusses various imaging techniques and their applications, highlighting characteristic radiological features of common postoperative complications. It underscores the importance of a multidisciplinary approach, emphasizing close collaboration between radiologists and surgeons to optimize surgical decision-making and improve patient management post-pancreatic surgery.

## 1. Introduction

Pancreatic ductal adenocarcinomas (PDACs), but also benign cystic lesions and chronic pancreatitis, require pancreatic surgery entailing a number of intricate operations [1,2,3]. These procedures necessitate the skills of highly qualified surgeons and drastically change the structure of the gastrointestinal tract [4,5]. Pancreatic surgery continues to be linked to significant postoperative problems, morbidity, and mortality even when it is carried out in high-volume hospitals [6,7,8].

The four distinct forms of pancreatectomy are pancreaticoduodenectomy (PD), distal pancreatectomy (DP), central pancreatectomy (CP), and total pancreatectomy (TP), each of which is designed to address specific indications [9,10,11].

For clarity and ease of reference, Table 1 summarizes the main types of pancreatic surgery, including their indications, resected structures, and surgical approaches [9,10,11].

The usual surgical method for resectable pancreatic head PDAC and other periampullary cancers, such as duodenal tumors, ampullary adenocarcinoma, and distal bile duct cholangiocarcinoma, is PD, also referred to as the Whipple surgery [12,13,14]. Pancreatic neuroendocrine tumors (NETs), gastrointestinal stromal tumors (GISTs), mucinous cystic neoplasms, isolated metastatic lesions, chronic pancreatitis with an inflammatory mass in the pancreatic head, and severe pancreatic damage are additional grounds for PD [15,16,17,18].

Pancreaticoduodenectomy (PD), or the Whipple procedure, remains the cornerstone of treatment for malignant and select benign conditions of the pancreatic head and periampullary region. Despite advances in surgical techniques and perioperative care, PD is associated with considerable morbidity, with complication rates approaching 50% in high-volume centers, and mortality rates ranging from 1–5% depending on institutional expertise. The conventional PD technique involves the resection of the pancreatic head, distal bile duct, gallbladder, duodenum, proximal jejunum, and part of the stomach [19,20,21,22]. Gastrointestinal continuity is restored through gastrojejunostomy, while pancreatic and biliary drainage is ensured via pancreaticojejunostomy and choledochojejunostomy [23,24]. Over time, modifications of PD have emerged, with the most common being pylorus-preserving pancreaticoduodenectomy (PPPD), in which the stomach is spared by transecting the duodenum just beyond the pylorus, followed by duodenojejunostomy [25,26].

When cancers spread from the pancreatic head into the left pancreas, when there are recurring tumors in the pancreatic remnant, or when obtaining a negative resection margin in pancreatic head cancer is not practical, TP is usually taken into consideration [27,28,29]. Other indications include numerous pancreatic metastases, multifocal NETs, anastomotic leakage with sepsis, bleeding following PD, and multifocal intraductal papillary mucinous neoplasms (IPMNs) with high-risk characteristics affecting the whole gland [8,30,31].

DP is primarily indicated for lesions in the pancreatic body and tail, including tumors, chronic pancreatitis, trauma, and arteriovenous malformations affecting these regions [32,33,34]. This procedure can be performed via open surgery or a laparoscopic approach. DP entails resection of the pancreas to the left of the superior mesenteric vein/portal vein axis, with the transection line determined by the lesion’s location [35,36,37]. Unlike PD, DP does not involve resection of the duodenum or distal bile duct. In most cases, DP is accompanied by splenectomy; however, spleen-preserving techniques may be employed depending on the extent of the disease [38,39,40].

CP serves as an alternative to DP for treating benign or low-grade malignant lesions in the pancreatic neck and body. This approach aims to conserve pancreatic parenchyma, thereby minimizing the risk of postoperative endocrine and exocrine insufficiency [41,42].

When less intrusive treatments, including percutaneous drainage, are unsuccessful, pancreatic necrosectomy (PN), a therapy option for patients with severe acute necrotizing pancreatitis exacerbated by infected necrosis, may be considered [24,43,44,45]. Percutaneous, laparoscopic, and endoscopic procedures are examples of minimally invasive techniques that can be used for PN in addition to the more conventional open surgical approach [46].

Major complications include postoperative pancreatic fistula (POPF), delayed gastric emptying, biliary leakage, and postoperative hemorrhage, all of which can significantly affect patient outcomes and recovery [47,48]. Pancreaticoduodenectomy (PD) has the highest risk of serious complications among these surgeries [18,20,21,22,23]. Morbidity is still considerable, impacting around 50% of patients, even though improvements in surgical methods and perioperative care have greatly decreased the death rate, which is today estimated to be around 1% in high-volume hospitals [9,49,50,51,52].

Imaging plays a pivotal role in the early identification and management of these complications. Among available modalities, contrast-enhanced computed tomography (CECT) is the first-line imaging tool due to its accessibility, rapid acquisition, and capacity to delineate surgical anatomy and complications. Magnetic resonance imaging (MRI), including magnetic resonance cholangiopancreatography (MRCP), complements CT with superior soft-tissue contrast and ductal system evaluation, particularly when hepatobiliary contrast agents are employed [53,54,55,56,57]. Accurately interpreting imaging results, which have a big impact on patient outcomes, requires a deep grasp of post-surgical anatomy and associated problems.

This narrative review aims to synthesize current imaging strategies used after PD, emphasizing the radiological features of key postoperative complications. In doing so, it seeks to enhance the multidisciplinary dialogue between radiologists and surgeons, ensuring timely and precise interpretation of findings in the postoperative setting.

## 2. Imaging Modalities

### 2.1. Computed Tomography

The most widely used imaging technique for assessing patients after pancreatic surgery is contrast-enhanced computed tomography (CECT) [58,59].

CECT is recommended in the postoperative context because of its quick acquisition, excellent spatial and contrast resolution, and potent diagnostic capabilities in detecting both early and late problems related to pancreatic surgery [60,61]. It also clearly defines postoperative anatomy, which makes it easier to locate anastomotic sites.

A comprehensive CECT protocol should include an unenhanced phase, which is valuable for detecting hyperattenuating components such as blood products, surgical clips, and other postoperative materials [12,62,63,64]. This phase also aids in distinguishing lesions that exhibit contrast enhancement in later phases. Following the unenhanced acquisition, the pancreatic arterial phase is essential. This phase is optimally obtained using a bolus-triggering (BT) technique, with contrast administration timed to reach a threshold of approximately 120 HU in the abdominal aorta, typically with an 18 s delay [65,66,67,68]. The pancreatic arterial phase permits early diagnosis of problems, such as active bleeding along the surgical margins, and guarantees appropriate augmentation of the remaining pancreatic tissue [69,70]. The portal venous phase, which is obtained utilizing BT methods about 70 s after crossing the contrast threshold, is the next important phase [71,72]. Abdominal organs show their distinctive enhancement during this phase, increasing the diagnostic precision for both pancreatic and extra-pancreatic problems. The portal venous phase is especially useful for detecting secondary changes in nearby organs, such as the liver and spleen, and for examining peri-pancreatic infections, such as abscess formation or walled-off necrosis [73,74,75,76]. It also offers vital information regarding the primary abdominal venous structures’ patency. Since a delayed phase makes it easier to see hemorrhagic foci close to the surgical site, it may provide additional diagnostic information, especially in suspected cases of slow-flow bleeding. The need for this phase should be carefully evaluated, though, particularly in light of the “As Low As Reasonably Achievable” (ALARA) principle, which aims to reduce radiation exposure [77,78]. The delayed phase’s clinical usefulness is still up for question, and there are not many studies to back up its regular use, so its use is case-specific [79,80,81]. To enhance communication between radiologists and surgeons, multiplanar reconstructions (MPRs), particularly in coronal and sagittal planes, are highly recommended. These views are especially useful for surgeons, who often rely on non-axial perspectives during intraoperative assessments.

### 2.2. Magnetic Resonance Imaging

Because of its high diagnostic accuracy and superior contrast resolution, magnetic resonance imaging (MRI) is still a useful technique for evaluating patients following pancreatic surgery [82,83,84,85]. However, before suggesting MRI in the postoperative context, a number of restrictions should be taken into account. One major drawback is the requirement for breath-holding during image acquisition, which can be challenging for patients in the early postoperative period, especially those with pulmonary or pleural complications. Motion artifacts caused by respiration can significantly impact image quality and reduce diagnostic reliability.

Another limitation of MRI is its higher cost compared to other imaging modalities, both in terms of financial resources and scan duration [86,87,88]. Although abbreviated MRI protocols have been proposed to address these concerns, time constraints remain an important consideration [89,90,91,92]. Notwithstanding these drawbacks, MRI is still regarded as the gold standard for assessing anastomotic integrity and the biliary and pancreatic ductal systems. Magnetic resonance cholangiopancreatography (MRCP), which makes use of highly T2-weighted imaging (T2WI) sequences obtained using a variety of technical techniques, is primarily responsible for its improved diagnostic capabilities. The biliary system, pancreatic ducts, and surgical anastomoses may all be seen in great detail with MRCP, which makes it especially helpful for identifying difficulties after surgery.

Diffusion-weighted imaging (DWI), multiplanar T1- and T2-weighted imaging with and without fat saturation, and at least one 3D MRCP acquisition should all be included in a thorough MRI procedure for post-pancreatic surgical evaluation. When clinically indicated, contrast media can be administered to enhance diagnostic capabilities [93,94,95]. Extracellular contrast agents (ECAs) are particularly useful for assessing pancreatic and peri-pancreatic complications, with dynamic contrast-enhanced sequences following a protocol similar to that used in CECT. Hepatobiliary contrast agents (HBAs) offer extra diagnostic value for suspected biliary or hepatic problems, especially in identifying bile leakage. For the best evaluation of the biliary system and choledochojejunostomy, the hepatobiliary phase should be obtained at around 90 min post-injection for gadobenate dimeglumine (Gd-BOPTA) or 20 min for gadoxetate disodium (Gd-EOB-DTPA), depending on the particular agent used.

## 3. Biliary Leakage

**Figure 1:**
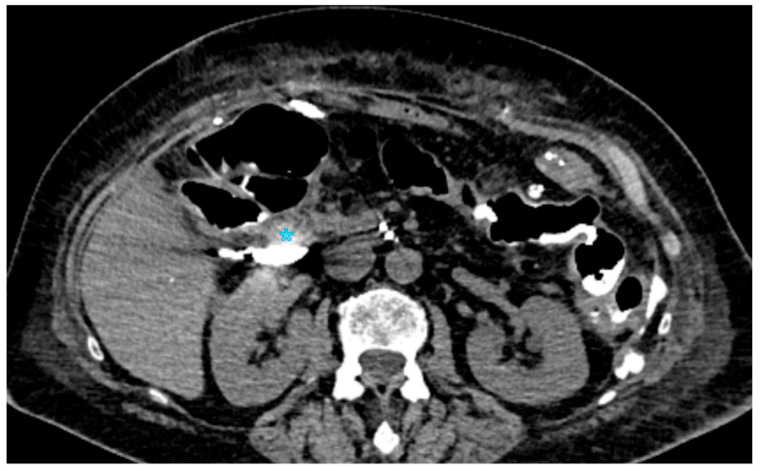
CT cholangiography through a Kehr tube showing the spread of contrast medium (blue asterisk) in the subhepatic region, anterior to the kidney, compatible with a biliary leak in a patient who underwent duodenopancreatectomy 5 days earlier.

The International Study Group of Liver Surgery (ISGLS) defines biliary leakage after pancreaticoduodenectomy as a drain fluid bilirubin concentration that is at least three times higher than plasma bilirubin levels, measured on or after the third postoperative day [96,97]. This condition can occur in as many as 8% of cases [16,20,98]. Although biliary fistulas are less frequent than postoperative pancreatic fistulas and are linked to a reduced risk of complications and death, they can nevertheless result in secondary complications, especially the formation of an intra-abdominal abscess, which may call for further treatments [21,41,99,100]. The primary risk factors for biliary leakage include excessive dissection of the hepatic duct (“skeletonization”), which can compromise vascular supply and healing; a small bile duct diameter, which increases anastomotic tension and impairs healing potential; and anastomosis involving the common bile duct, which presents technical challenges in maintaining an adequate seal [62,101,102,103]. To minimize these risks, several protective strategies have been developed, including refined reconstruction techniques to enhance anastomotic integrity, percutaneous biliary drainage to divert bile and promote fistula closure, and intraoperative T-tube placement to provide controlled bile drainage and reduce anastomotic stress [29,104,105,106,107]. In cases of biliary leakage, fluid collections are typically observed near the choledochojejunostomy, requiring cross-sectional imaging for accurate assessment [108,109,110]. On contrast-enhanced computed tomography (CECT), biliary leakage appears as homogeneously hypoattenuating fluid collections, generally without a well-defined capsule [4,46,111,112] (Figure 1).

On MRI, T1-weighted imaging (T1WI) demonstrates hypointense collections, while T2-weighted imaging (T2WI) shows hyperintense fluid accumulations, consistent with bile extravasation [113,114]. One of the main diagnostic challenges in imaging biliary leakage is differentiating it from a pancreatic fistula, given the close anatomical relationship between the choledochojejunostomy and pancreaticojejunostomy. Since both complications can present with fluid collections in similar locations, clinical correlation and biliary-specific imaging techniques are crucial for accurate diagnosis. Magnetic resonance cholangiopancreatography (MRCP) with HBAs can help in distinguishing these conditions [115,116]. By administering HBAs and acquiring images during the hepatobiliary phase (HBP), it is possible to directly visualize active bile leakage, as the contrast extravasates into the fluid collection, confirming a biliary origin HBA [117]. Since HBAs do not fill the pancreatic duct, the absence of contrast in the pancreatic region further differentiates it from a pancreatic fistula [48,118]. Studies have demonstrated that HBP-enhanced MRI using Gd-EOB-DTPA, when combined with MRCP, significantly improves diagnostic accuracy in detecting biliary leaks [119,120]. Compared to MRCP alone, this combined approach has shown an accuracy of 84%, a specificity of 100%, and a statistically significant improvement (*p* < 0.05) in diagnostic performance [121,122,123]. These findings emphasize the importance of HBP-enhanced MRI in optimizing the detection and characterization of biliary leaks, thereby improving the management of postoperative complications.

## 4. Fluid Collections and Abscesses

Up to 30% of patients having pancreatic resections experience fluid collections and the development of an abscess in the upper abdomen, which are frequent post-pancreatic surgical sequelae [124,125,126]. These collections typically arise due to postoperative pancreatic leaks, most commonly from pancreatic fistulae or anastomotic dehiscence at the pancreaticojejunostomy or choledochojejunostomy after pancreaticoduodenectomy. While some postoperative fluid collections resolve spontaneously, others may evolve into infected abscesses, leading to increased morbidity, secondary septic complications, and frequent hospital readmissions. Differentiating between benign postoperative collections and infected abscesses is crucial, as the latter require targeted interventions [127]. For larger or symptomatic fluid collections, percutaneous drainage remains the preferred treatment to effectively manage infections and prevent further complications.

The progression of postoperative fluid collections follows a predictable pattern. Acute fluid accumulations and pancreatic necrosis typically occur within the first week after surgery. By the second to third week, infected necrosis becomes more common, while by the fourth week or later, these collections may transform into pseudocysts or pancreatic abscesses. Identification and characterization of these collections are mostly dependent on cross-sectional imaging, especially contrast-enhanced computed tomography (CECT) and magnetic resonance imaging (MRI).

On CECT, acute fluid collections appear as hypoattenuating areas near the surgical site, often irregular in shape and located adjacent to the pancreatic remnant, anastomotic sites, or nearby vascular structures. In the early stages, these collections are confined by the abdominal fascia and typically lack a discernible capsule.

**Figure 2:**
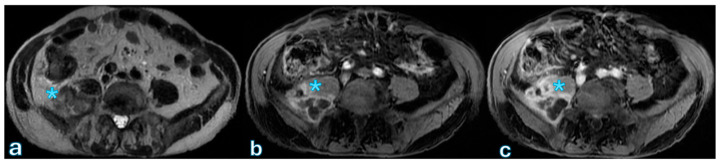
The MR T2-weighted image sequences (**a**), T1-weighted sequence after administration of contrast-enhanced arterial phase (**b**), and late phase (**c**) reveal a psoas muscle lesion with peripheral enhancement, which is consistent with an abscess indicated with blue asterisk. This finding was observed in a patient with fever and abdominal pain, 1 month after surgery for pancreatic adenocarcinoma localized to the body.

On MRI, particularly T2-weighted imaging with fat saturation, fluid collections appear hyperintense, though their signal may be inhomogeneous if proteins or hemorrhagic components are present. Hypointense regions within the collection may correspond to blood degradation products, indicating a more complex composition (Figure 2).

In cases of pancreatic necrosis, CECT reveals heterogeneous attenuation due to the presence of nonviable tissue, fat, and hemorrhagic debris [128]. On T2-weighted MRI, necrotic regions appear inhomogeneous, reflecting the mixed composition of necrotic debris and surrounding inflammatory changes. Pancreatic necrosis is often partially or completely encapsulated, forming a well-defined peripheral rim with contrast enhancement on both CT and MRI, suggesting organizing necrosis or early wall formation [73,129]. If the necrotic collection becomes superinfected, the presence of air bubbles within the collection becomes a key diagnostic feature. On CT, air bubbles appear as low-density foci, while on MRI, air appears as signal voids across all sequences, confirming the presence of infection.

Pseudocyst development and pancreatic abscesses are examples of late consequences. On both CT and MRI, pancreatic abscesses appear as fluid collections with a thicker, enhancing wall. The diagnosis is confirmed by the presence of intralesional air, which is pathognomonic for infection. Conversely, pseudocysts are spherical, well-circumscribed fluid collections that seem uniformly hypoattenuating on CT. They are easy to distinguish from solid or necrotic lesions on MRI because they appear hypointense on T1-weighted imaging and uniformly hyperintense on T2-weighted imaging. Debris from the pseudocyst can occasionally be seen at the bottom of the sample as hypointense, irregular material.

Recognizing these imaging features is critical for distinguishing between sterile and infected postoperative collections, allowing for appropriate management decisions. Depending on the severity and progression of the collection, treatment may involve conservative management, image-guided percutaneous drainage, or surgical intervention to optimize patient outcomes.

## 5. Pancreatitis

Postpancreatectomy Acute Pancreatitis (PPAP) is an increasingly recognized complication following pancreatic resection, defined by the International Study Group of Pancreatic Surgery (ISGPS) as an acute inflammatory process affecting the remnant pancreatic parenchyma within the first three postoperative days after partial pancreatectomy [130]. While the full clinical implications of PPAP are still under investigation, it has been identified as a significant contributor to secondary complications, particularly postoperative pancreatic fistula (POPF) [131,132,133].

Three essential criteria must be met in order to diagnose PPAP: clinical deterioration, which includes signs of systemic impact or worsening postoperative status; radiologic findings that indicate pancreatic inflammation; and persistent postoperative hyperamylasemia, which is defined by elevated serum amylase levels that surpass the institutional upper limit for at least 48 h after surgery [130].

It can be difficult to diagnose PPAP radiologically because postoperative inflammatory alterations frequently resemble its imaging presentation.

However, fat stranding close to the surgery site, focal or diffuse pancreatic enlargement, and inhomogeneous parenchymal enhancement are important imaging characteristics of contrast-enhanced CT (CECT) and MRI, which offer useful diagnostic insights.

In order to distinguish hemorrhagic pancreatitis from straightforward postoperative edema, hemorrhagic components may also be present. These can manifest as hyperintense regions on pre-contrast T1-weighted MRI or hyperattenuating areas on unenhanced CT.

Diffusion-weighted imaging (DWI), which provides functional information on tissue properties, considerably improves the diagnosis accuracy of PPAP. Increased cellularity and interstitial edema are indicative of pancreatic inflammation, as is a hyperintense signal on DWI with decreased apparent diffusion coefficient (ADC) values. By helping to differentiate between active inflammation and typical postoperative alterations, this imaging feature lowers the chance of serious complications like POPF by enabling early detection and prompt action.

## 6. Postoperative Hemorrhage

One of the most life-threatening side effects after pancreatic resections is postoperative hemorrhage (PPH), which has a 2% to 8% incidence and a 10% to 38% death rate [134]. PPH is categorized by the International Study Group for Pancreatic Surgery (ISGPS) according to three factors: location (intraluminal or extraluminal), timing (early or delayed), and severity (mild or severe) [135]. While delayed post-pancreatectomy hemorrhage (DPH), which is more common and has a mortality rate of 30% to 50%, is typically caused by complications like anastomotic leakage, pancreatic fistula, infection, or intra-abdominal abscess, early post-pancreatectomy hemorrhage (EPH) happens within the first 24 h after surgery and is usually caused by inadequate intraoperative hemostasis [135].

Particularly in cases with pancreatic fistulas, vascular structures such as the gastroduodenal artery stump may be exposed to digestive enzymes, which may cause gradual erosion of the vessels and consequent bleeding [136,137]. A hemoglobin reduction of less than 3 g/dL and no clinical consequence are considered moderate bleeding, while hemodynamic instability and a hemoglobin drop of at least 3 g/dL are considered severe bleeding. The majority of DPH cases start in the arteries and frequently manifest as “sentinel bleeding”, which is a small hemorrhagic episode that is discovered by abdominal drains or nasogastric tubes prior to a big hemorrhagic event [138]. The patient may be guided toward prompt endovascular or surgical intervention, perhaps preventing catastrophic bleeding, if this early warning sign is recognized.

The gastroduodenal artery stump is the most typically affected region, followed by the splenic artery, inferior pancreaticoduodenal artery, splenic vein stump, and intrapancreatic arteries [139]. From a radiological point of view, PPH most commonly affects peripancreatic vascular structures. Anastomotic suture lines, such as those at the pancreatic-enteric, duodeno-enteric, jejuno-jejunal, and gastro-enteric anastomoses, as well as the resection site or a ruptured pseudoaneurysm, are other possible causes of bleeding.

PPH can be categorized as intraluminal, which originates from anastomotic sites, pancreatic surfaces, stress ulcers, or hemobilia, or extraluminal, which occurs within the abdominal cavity due to vascular injury, diffuse bleeding from the resection site, or pseudoaneurysm rupture. In hemodynamically unstable patients, direct angiography is the preferred imaging modality as it allows for immediate identification of the bleeding source and enables endovascular treatment. For stable patients with suspected DPH, contrast-enhanced computed tomography (CECT) is the first-line imaging technique, offering near-complete accuracy in detecting hemorrhage while also providing critical details about vascular anatomy, anatomical variations, and vessel patency to guide selective endovascular interventions.

A comprehensive CECT protocol for postoperative bleeding includes an unenhanced phase to identify hyperattenuating hemoperitoneum and differentiate pre-existing surgical materials from bleeding foci. The arterial phase, acquired after high-flow intravenous contrast administration (3.5–4 mL/s), is crucial for detecting active contrast extravasation, evaluating vascular anatomy, and identifying pseudoaneurysms. The portal venous phase, performed 70–90 s post-injection, assesses progressive arterial extravasation and potential venous bleeding, while the delayed phase, acquired 140 s to 5 min post-contrast, is particularly useful in patients with impaired cardiac function to confirm slow-flow extravasation. A key CT finding indicative of active postoperative bleeding is contrast extravasation outside vascular structures, appearing as an irregular hyperattenuating focus (approximately 90 HU) in the arterial phase that is absent on non-contrast images. During later stages, this hyperdensity usually enlarges and changes in shape, indicating ongoing bleeding. Contrast extravasation in intraluminal hemorrhage situations may appear irregular because of peristaltic motion or settle in a dependent position within the intestinal lumen. The “sentinel clot sign,” which is another crucial radiologic indicator, is a hyperdense clot seen close to the bleeding site, indicating a recent hemorrhagic event that may have temporarily stopped [140]. These imaging characteristics are essential for a timely and precise diagnosis, which enables the right endovascular or surgical intervention to avoid serious consequences.

## 7. Postoperative Pancreatic Fistula

With an incidence ranging from 13% to 41%, postoperative pancreatic fistula (POPF) is the most common complication after pancreatic surgery and a major contributor to morbidity and mortality [141]. It is characterized by a detectable drain output that happens on or after the third postoperative day and an amylase concentration that is more than three times the typical serum level [20,22,41]. POPF can result in intra-abdominal abscesses and, in extreme situations, subsequent bleeding. It is caused by the leakage of pancreatic secretions at an anastomotic site or surgically removed surface. A tiny pancreatic duct, soft pancreatic texture, high-risk underlying disease, and substantial intraoperative blood loss are some of the variables that predispose individuals to POPF. Three grades of POPF are distinguished by the International Study Group of Pancreatic Fistula (ISGPF) [20]: Grade A, which is a biochemical leak without clinical symptoms; Grade B, which is defined by persistent drainage that lasts longer than three weeks and necessitates additional interventions like surgical or percutaneous drainage or presents complications like bleeding or infection; and Grade C, which requires reoperation and is linked to organ failure or death. For the purpose of directing management tactics and reducing serious difficulties, accurate classification is crucial.

Although the best time and indications for postoperative CT are still unknown, contrast-enhanced computed tomography (CECT) is the principal imaging modality used to diagnose POPF. The incidence of serious complications does not seem to be considerably impacted by routine early CT imaging during the first postoperative week. A structured methodology for early complication detection was recently presented by the PORSCH experiment [142]. It suggests CT scans based on clinical and laboratory indicators, including physical examination, changes in drain output, and blood test variations. According to a retrospective investigation, CECT on the seventh postoperative day provided an 83% specificity and 63% sensitivity for identifying POPF. Fluid collections close to the pancreaticojejunostomy site or resection margin, as well as obvious anastomotic disruption, are imaging characteristics diagnostic with Grade B or C POPF. The diagnosis is further supported by certain patients that exhibit aberrant connection with the main pancreatic duct.

**Figure 3:**
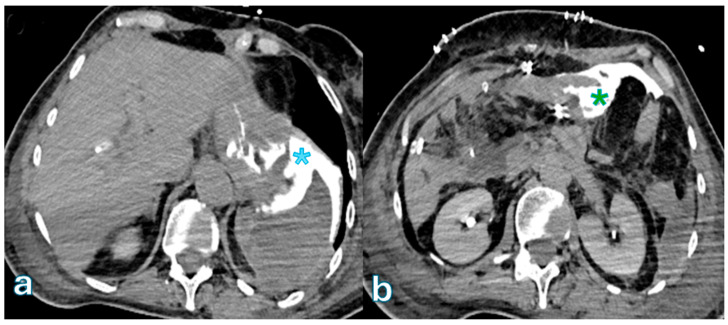
Axial CT with oral-administrated iodinated contrast showing the spread of extraluminal water-soluble contrast medium in a patient who underwent duodenopancreatectomy 3 days earlier. Oral contrast leakage is observed at the level of the gastrojejunostomy ((**b**) green asterisk), with distribution in the perisplenic region ((**a**), blue asterisk). These findings are indicative of gastrojejunostomy anastomosis dehiscence.

Key CT features that differentiate severe (Grade C) POPF from milder cases (Grades A/B) were identified in a study by Lee et al. [143] that involved 235 postoperative patients. These features included acute necrotic collections, which indicated a more severe inflammatory process; pancreaticojejunostomy defects, which indicated incomplete or failed anastomosis; and pancreaticojejunostomy dehiscence, which is defined as a separation of >2 mm between the pancreatic duct and jejunal mucosa (Figure 3).

Secondary complications such as bleeding, pancreatitis, abscess development, and sepsis can also result with POPF; for proper clinical therapy, these conditions need to be carefully assessed on post-contrast CT [48,144]. The type of surgical treatment, the nature of the pancreatic lesion, the postoperative course, and individual patient factors are among the risk factors for postoperative pancreatic fistula that have been discovered. With an emphasis on body composition metrics and pancreatic parenchymal characteristics, recent research has examined preoperative CECT data to forecast POPF risk. Increased pancreatic gland thickness, pancreatic texture and borders, and a larger main pancreatic duct width are specific imaging markers linked to an increased risk of clinically severe POPF. Because pancreatic attenuation values may indicate underlying parenchymal fibrosis, they have also been linked to POPF risk. Furthermore, sarcopenia and visceral obesity have been associated with a higher risk of POPF, indicating that postoperative outcomes may be influenced by muscle and metabolic state [145,146]. By identifying these preoperative imaging indicators, surgical planning can be improved and risk can be stratified, which will ultimately lower the frequency and severity of postoperative pancreatic fistula.

## 8. Anastomotic Strictures

Postoperative biliary anastomotic strictures are a relatively uncommon complication following pancreaticoduodenectomy (PD) but can significantly impact long-term outcomes [147]. These strictures often present later in the postoperative course and are typically identified during oncologic follow-up due to the onset of recurrent cholangitis [148]. Pathogenesis is multifactorial, with key risk factors including a small bile duct diameter, which increases anastomotic tension; ischemia at the anastomosis, which impairs healing and leads to fibrotic stricture formation; and reflux of gastric or enteric contents, which may trigger secondary cholangitis and further anastomotic narrowing [149].

Management strategies for biliary strictures depend on the severity and response to initial therapy. Non-surgical approaches, such as percutaneous transhepatic cholangiography (PTC) and endoscopic balloon dilation, can provide short-term symptomatic relief, particularly in patients unfit for surgery. However, when conservative management fails, surgical revision via Roux-en-Y hepaticojejunostomy is the preferred approach, ensuring long-term biliary drainage and reducing the risk of recurrent strictures and cholangitis.

## 9. Hepatic Complications

### 9.1. Steatosis

About 2.2% of patients had a postoperative increase in liver enzymes after pancreatic resection; this is usually seen as a temporary and typical reaction [7,10]. Serum aminotransferase levels usually reach their maximum on the first postoperative day and then progressively go back to normal over the course of five days [150]. However, with an incidence of up to 37% between 4 and 12 months postoperatively, de novo Non-Alcoholic Fatty Liver Disease (NAFLD) has become a recognized long-term consequence [151].

Two main factors are responsible for the development of postoperative hepatic steatosis: pancreatic exocrine insufficiency with malabsorption, which results in deficiencies of amino acids, insulin, carnitine, and choline, all of which impair lipid metabolism; and metabolic syndrome and insulin resistance, which raise circulating free fatty acids, hepatic fat uptake, and lipogenesis. The surgical inflammatory response and metabolic changes brought on by chemotherapy are additional significant factors.

The link between de novo Non-Alcoholic Fatty Liver Disease (NAFLD)/Non-Alcoholic Steatohepatitis (NASH) and pancreatic exocrine insufficiency is supported by clinical research. According to Tanaka et al., patients with post-pancreaticoduodenectomy (PD) NASH exhibited lower levels of apolipoprotein B, serum albumin, cholesterol, and Body Mass Index than those with conventional NASH [152]. This suggests that the main underlying cause of PD NASH is pancreatic exocrine dysfunction. While conventional NAFLD risk factors like obesity, hyperlipidemia, insulin resistance, and chemotherapy do not seem to significantly contribute to de novo NAFLD following pancreatic surgery, Nakagawa et al. further confirmed that postoperative pancreatic exocrine insufficiency is the only independent risk factor for NAFLD.

**Figure 4:**
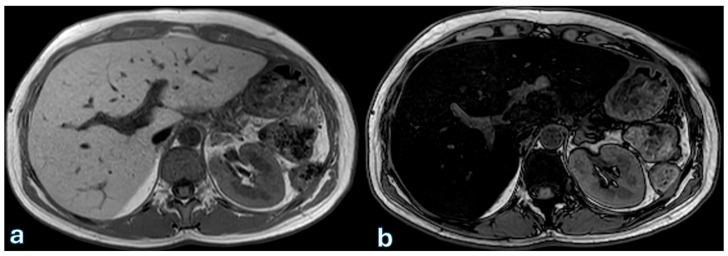
MR T1-weighted image sequences acquired in-phase (**a**) and out-of-phase (**b**) showing a marked signal drop between the in-phase and out-of-phase sequences, consistent with diffuse hepatic steatosis. This finding was observed in a patient at the first follow-up, conducted one month after surgery for pancreatic adenocarcinoma located in the head.

**Figure 5:**
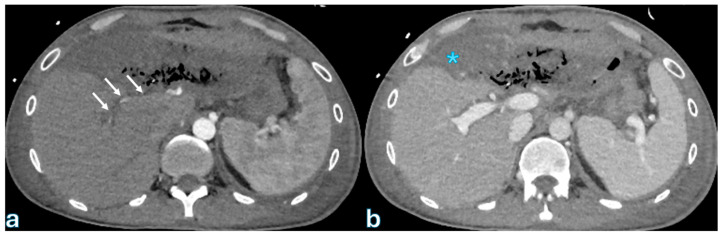
CT angiography of the abdominal vessels in (**a**) arterial and (**b**) portal venous phase showing nonopacification of the common hepatic artery (white arrows) and left portal branch, with subsequent ischemia of the corresponding liver parenchyma (blue asterisk). This finding was observed in a patient who underwent major pancreatic resection for pancreatic head adenocarcinoma four days after surgery and is consistent with embolization-induced liver infarction.

Detecting and quantifying hepatic steatosis through imaging is clinically relevant. While ultrasound (US) can provide qualitative detection, it lacks accuracy for precise quantification [153]. Computed tomography (CT) provides objective attenuation measurements, though its sensitivity varies [154]. Magnetic resonance imaging (MRI), particularly proton density fat fraction (MRI-PDFF), remains the most accurate method for detecting and quantifying hepatic steatosis, though its high cost and limited availability restrict its widespread use (Figure 4 and Figure 5) [155].

When absolute liver attenuation is ≤40 HU or pre-contrast liver attenuation is at least 10 HU lower than spleen attenuation, significant hepatic steatosis may be indicated on CT. Mild-moderate steatosis is defined as liver attenuation between 40.0 and 48.6 HU, mild steatosis as attenuation between 48.6 and 57.2 HU, and normal liver as attenuation ≥ 57.2 HU, according to a CT-based grading system connected to MRI-PDFF estimates.

A post-contrast liver attenuation threshold of <90 HU increases sensitivity to 90.5% but decreases specificity to 78.4%. On contrast-enhanced CT (CECT), mild steatosis is diagnosed when post-contrast liver attenuation is <80 HU, with a sensitivity of 77.8% and specificity of 93.2%. Although CT is useful, it is not regarded as a screening method for radiation-induced hepatic steatosis; however, its evaluation should be taken into account anytime CT is done for other purposes after pancreatic surgery.

Beyond qualitative and quantitative liver parenchyma assessment, additional imaging findings, such as atrophy of the pancreatic remnant, have been linked to the development of de novo NAFLD. Studies have shown that NAFLD is more prevalent in patients with a small pancreatic remnant volume (<10 mL) at one month postoperatively, as measured on CT.

### 9.2. Hepatic Infarction

Hepatic infarction is a rare but serious complication that can result from global hepatic hypoperfusion due to intraoperative systemic hypotension, which is usually reversible, or vascular occlusion involving hepatic arteries, which may lead to irreversible ischemia and necrosis [7].

After pancreatic surgery, hepatic infarction is not common. Six out of 545 patients having PD experienced hepatic ischemia problems in research by Gaujoux et al., four of which cases were directly linked to intraoperative hepatic artery injury [156]. Transaminase levels increase 24 h after surgery in cases of hepatic perfusion failure; mild-to-moderate cases have a brief elevation of the enzyme that returns to normal in two weeks. In extreme situations, an enzyme increase that lasts longer than ten days indicates irreparable liver damage.

Early detection of hepatic perfusion failure is critical for guiding timely interventions. Management may include endovascular revascularization by interventional radiology or surgical revascularization, depending on the severity of the infarction and the underlying vascular pathology.

**Figure 6:**
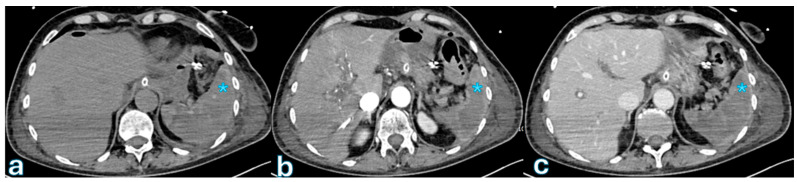
Non-contrast CT image (**a**) and post-contrast CT images with iodinated contrast in the arterial (**b**) and portal (**c**) phases, showing marked and persistent mottled opacification of the splenic artery, with subsequent lack of opacification in the downstream parenchyma (blue asterisk). This finding was observed in a patient who underwent major pancreatic resection for adenocarcinoma of the body of the pancreas one week prior and is consistent with embolic occlusion of the splenic artery, resulting in splenic infarction.

To assess hepatic perfusion anomalies and detect vascular pathology, contrast-enhanced CT (CECT) and MRI (containing arterial and portal venous phases) are essential. Wedge-shaped regions of decreased enhancement, which are usually well-defined and free of mass effect, are imaging characteristics of hepatic infarction (Figure 6).

Infarcted areas show up as slightly hyperintense on MRI T2-weighted imaging (T2WI), which is indicative of ischemia or edematous alterations.

In addition to identifying infarction, CT and MRI can detect underlying vascular abnormalities that cause hepatic ischemia, such as portal vein thrombosis, hepatic artery thrombosis, and hepatic or celiac artery injury/dissection. Furthermore, patients may be at risk for postoperative hepatic infarction if they have pre-existing vascular disorders such as mesenteric vasculitis, median arcuate ligament syndrome, fibromuscular dysplasia, or significant atherosclerotic disease, especially near the celiac artery origin. Preoperative vascular imaging should be thoroughly examined to identify high-risk patients and foresee probable postoperative problems due to the possibility of hemodynamic instability or infection.

## 10. Splenic Complications

Following spleen-preserving distal pancreatectomy, the most common splenic complications include splenic infarction and perigastric collateral formation due to left-sided portal hypertension [38]. Splenic infarction may lead to a gradual reduction in splenic volume, but in most cases, patients retain at least 40% of their splenic volume after six months, without requiring re-intervention [157,158]. Perigastric collateral vessels, which develop as a compensatory mechanism for altered splenic circulation, are generally asymptomatic, with no documented cases of intraluminal hemorrhage reported in the literature. Clinically, splenic infarction is often silent or presents with only mild abdominal pain, making diagnosis challenging [46,159].

**Figure 7:**
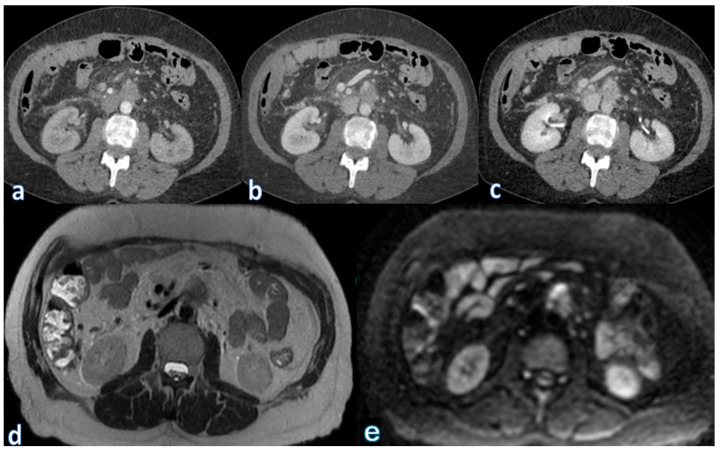
Axial CT images in the arterial (**a**), venous (**b**), and late (**c**) phases and T2-weighted MR (**d**) and DWI (**e**) images showing inhomogeneous solid tissue in the periaortic area as if resulting from local recurrence. This finding was observed in a patient who underwent major pancreatic resection for adenocarcinoma of the head of the pancreas three months after surgery.

On contrast-enhanced imaging, splenic infarction is radiologically represented as a wedge-shaped, non-enhancing area, with its base along the splenic capsule and its tip pointing toward the splenic hilum (Figure 7).

Over time, these infarcts may completely resolve or result in progressive splenic atrophy due to fibrotic contraction of the infarcted tissue. Early radiologic identification of splenic infarction is essential to ensure appropriate clinical monitoring while avoiding unnecessary interventions in asymptomatic patients.

## 11. Peculiar Issue After Pancreaticoduodenectomy: Disease Recurrence

**Figure 8:**
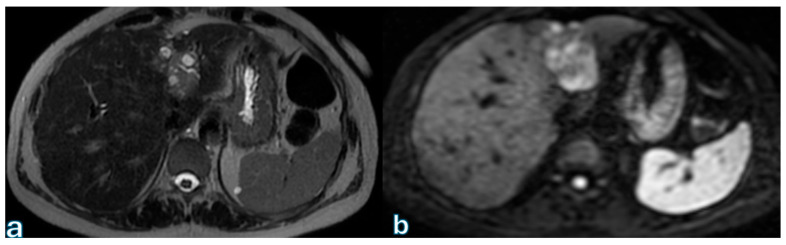
T2-weighted MR (**a**) and DWI (**b**) imaging sequences showing a secondary lesion at level IV of the liver segment. This finding was observed in a patient at the third follow-up, conducted approximately 9 months after surgery for pancreatic adenocarcinoma located in the head.

Local recurrence following curative-intent pancreatic cancer resection is common, typically occurring within two years postoperatively, with median survival significantly impacted by adjuvant therapy—ranging from 11 months with surgery alone to approximately 20 months when combined with chemotherapy [160]. Surveillance guidelines from the NCCN (v.2 2023) recommend clinical evaluation every 3–6 months for the first two years, including CA 19-9 monitoring and abdominal–pelvic CT imaging, although evidence supporting imaging frequency remains limited. Conversely, ESMO guidelines do not specify routine imaging protocols due to insufficient evidence on survival benefits [161]. Surveillance’s effectiveness in improving survival is controversial, with analyses like the SEER-Medicare database indicating no clear survival advantage. Nevertheless, early detection of isolated locoregional recurrence may permit re-resection, potentially enhancing survival outcomes. Recurrence patterns commonly involve local recurrence at surgical margins, particularly after pancreaticoduodenectomy, presenting as infiltrative soft tissue with perineural invasion and progressive vascular encasement, distinguishable from normal postoperative changes by serial imaging (Figure 8).

**Figure 9:**
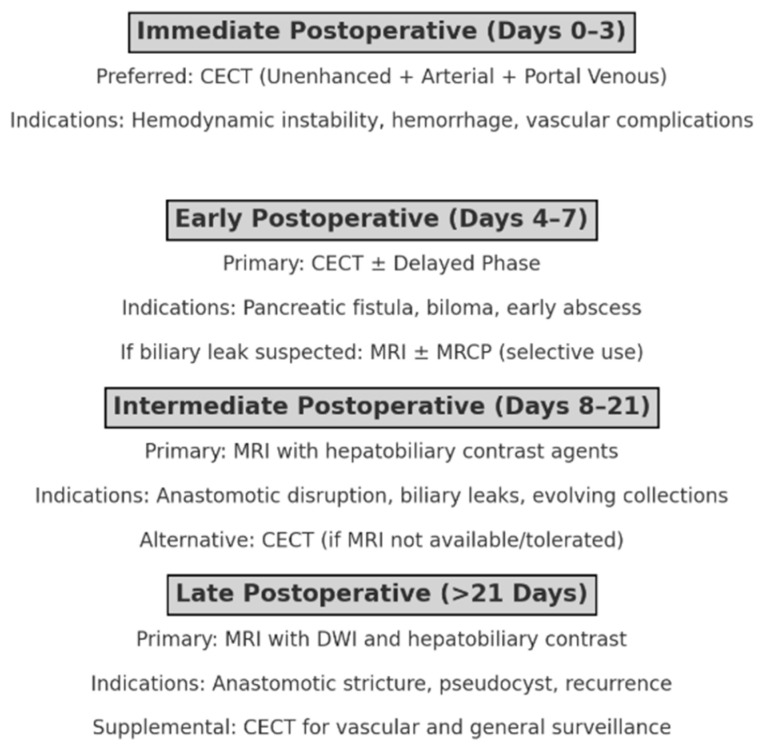
Time-stratified imaging algorithm for postoperative complication assessment. Imaging modality recommendations are based on postoperative day and clinical scenario. In the immediate period (days 0–3), contrast-enhanced CT (CECT) is preferred for detecting hemorrhage and vascular issues. During days 4–7, CECT remains the mainstay for identifying early fluid collections or leaks, with MRI considered in selective cases. Between days 8–21, MRI with hepatobiliary contrast agents is superior for evaluating biliary complications and anastomotic integrity. Beyond 21 days, MRI with diffusion-weighted imaging is optimal for detecting late complications or recurrence, while CECT is used for vascular and general surveillance.

A baseline postoperative scan at 6–8 weeks aids in accurate longitudinal assessment. Distant metastases frequently involve the liver and peritoneum, with MRI, especially diffusion-weighted and hepatobiliary contrast-enhanced imaging, demonstrating superior sensitivity in identifying subtle hepatic lesions compared to CT (Figure 9).

Lung metastases, while less common, suggest better prognosis when isolated, whereas skeletal and brain metastases remain rare. Given pancreatic cancer’s complex recurrence dynamics, employing a multimodal imaging strategy tailored to individual patient risk factors is crucial for optimal early detection and treatment guidance.

## 12. Imaging Strategy and Proposed Algorithm for Post-PD Complications

Radiologic imaging plays a pivotal role in the postoperative management of PD, offering essential diagnostic support in identifying complications such as pancreatic fistula, hemorrhage, abscesses, and biliary leakage. Among the available modalities, CECT remains the first-line imaging tool due to its widespread availability, rapid acquisition, and excellent delineation of both anatomy and early complications. MRI, including MRCP and HBAs, is increasingly recognized for its superior soft-tissue contrast and diagnostic specificity, especially in evaluating biliary leaks and subtle ductal pathologies.

The cost-effectiveness of postoperative imaging remains a debated topic, as it must balance the early detection of complications with healthcare resource utilization. While routine imaging may lead to earlier diagnosis of clinically silent complications, it can also result in increased costs and potential overtreatment. Conversely, a symptom-driven approach may delay detection, increasing morbidity. Therefore, guidance on the optimal timing of postoperative imaging—routine versus on-demand—should consider surgical complexity, complication risk profile, and institutional capabilities.

To address these limitations and guide optimal imaging decisions, we propose a pragmatic, time-stratified imaging algorithm based on postoperative day and clinical scenario. In the immediate postoperative period (days 0 to 3), CECT is the preferred modality, especially in the presence of hemodynamic instability or clinical signs suggestive of hemorrhage or vascular complications. The inclusion of unenhanced, arterial, and portal venous phases allows for the detection of early bleeding, surgical site hematoma, or ischemia. Between days 4 and 7, when complications such as postoperative pancreatic fistula, biloma, or early abscess formation may emerge, CECT remains the cornerstone modality, occasionally supplemented by delayed phases in cases of suspected slow-flow hemorrhage. In patients with signs of biliary leakage and inconclusive CT findings, limited MRI with or without MRCP may be selectively considered.

During the intermediate postoperative window (days 8 to 21), when complications such as anastomotic disruption, evolving collections, or biliary leaks become more prevalent, MRI with hepatobiliary contrast agents offers superior sensitivity and anatomical resolution, particularly for choledochojejunostomy evaluation and bile leak characterization. In patients intolerant to MRI or where resources are constrained, CECT remains a valid alternative. Beyond three weeks post-surgery, the focus shifts to detecting late complications such as anastomotic strictures, pseudocyst formation, or disease recurrence. In this phase, MRI—especially with diffusion-weighted imaging and hepatobiliary contrast—offers high diagnostic yield for evaluating liver metastases or subtle peritoneal disease, while CECT remains useful for broader surveillance and vascular assessment. A comprehensive graphical flowchart is provided (Figure 9).

This integrated, phase-specific strategy underscores the importance of tailoring imaging selection to both temporal evolution and clinical context. A structured, scenario-driven algorithm not only improves diagnostic accuracy but also facilitates timely therapeutic decisions and fosters effective communication between radiologists and surgical teams in the complex landscape of post-PD care.

To enhance the practical applicability of the proposed imaging framework, a concise summary table is provided below (Table 2) [8,9,10,11,12,19,20,21,26,47,48,49,51,56,57,85,87].

Recent advances in artificial intelligence (AI) offer promising opportunities to enhance postoperative imaging interpretation and early complication detection. As highlighted in a systematic review by Stam et al., AI-based algorithms, including machine learning and deep learning models, have shown potential to outperform traditional statistical methods in predicting postoperative complications across major abdominal surgeries, including pancreatic procedures [162]. Notably, AI models have demonstrated high accuracy and specificity in detecting complications such as postoperative pancreatic fistula, particularly when trained on robust, balanced datasets and validated externally. However, before routine implementation, these tools require rigorous validation and integration within clinical workflows to ensure reliability, minimize false positives, and optimize clinical decision-making.

## 13. Conclusions

When evaluating patients with pancreatic cancer who have had surgical resection, cross-sectional imaging is crucial. Because of its great spatial and contrast resolution, contrast-enhanced computed tomography (CECT) continues to be the first-choice imaging modality. This allows radiologists and surgeons to communicate effectively, enabling prompt identification and management of problems.

MRI offers improved soft-tissue contrast and is essential for identifying distant metastatic illness and late surgical sequelae, while being more expensive and time-consuming. Diffusion-weighted imaging (DWI) and hepatobiliary contrast-enhanced MR cholangiography are two advanced MRI techniques that significantly improve diagnostic accuracy, especially for hepatic metastases, pancreatic fistulas, and biliary leakage.

Ultimately, accurate interpretation of imaging results and directing patients toward the best therapeutic therapy depend on tight cooperation between radiologists and surgeons.

## Figures and Tables

**Table 1:** Summary of the main types of pancreatic surgery, including their clinical indications, resected anatomical structures, and preferred surgical approaches. This table is designed as a practical reference for non-specialists to understand the rationale and technical variations behind each procedure. Surgical decision-making depends on factors such as tumor location, lesion type, risk of malignancy, and anatomical considerations. Whenever feasible, parenchyma-sparing and minimally invasive techniques are preferred to reduce postoperative morbidity and preserve endocrine and exocrine function.

Surgical Procedure	Main Indications	Structures Resected	Surgical Approach
Pancreaticoduodenectomy (PD)(Whipple or PPPD)	Resectable pancreatic head adenocarcinoma (PDAC), periampullary tumors (distal cholangiocarcinoma, ampullary carcinoma, duodenal tumors), neuroendocrine tumors (NETs), GISTs, mucinous cystic neoplasms, chronic pancreatitis with head mass, severe trauma	Pancreatic head, distal bile duct, gallbladder, duodenum, proximal jejunum, ±gastric antrum (if PPPD: pylorus is preserved)	Open or laparoscopic; reconstruction via gastro-/duodenojejunostomy, pancreaticojejunostomy, choledochojejunostomy
Distal Pancreatectomy (DP)	Lesions in the body and tail of the pancreas, localized chronic pancreatitis, trauma, vascular malformations	Pancreatic body and/or tail ± spleen	Open or laparoscopic; with or without spleen preservation
Central Pancreatectomy (CP)	Benign or low-grade malignant lesions in the neck or central body of the pancreas	Central segment of pancreas; preserves both head and tail	Open or laparoscopic; parenchyma-sparing to reduce endocrine/exocrine insufficiency
Total Pancreatectomy (TP)	Multicentric IPMN with high-risk features, multifocal NETs, unresectable margins with partial resection, recurrence, extensive trauma, complications like infected leaks or hemorrhage	Entire pancreas, bile duct, duodenum, gallbladder, ±spleen and part of stomach	Open or laparoscopic; leads to complete endocrine and exocrine insufficiency
Pancreatic Necrosectomy (PN)	Infected pancreatic necrosis not responsive to less invasive drainage	Necrotic pancreatic tissue	Open, laparoscopic, or endoscopic; part of step-up approach for necrotizing pancreatitis

**Table 2:** Summary of the main postoperative complications following pancreaticoduodenectomy (PD), including estimated incidence, preferred imaging modality, pathognomonic radiologic signs, and recommended acquisition phase. This table is intended as a practical reference to support radiologic interpretation and guide clinical management across different postoperative scenarios.

Complication	Incidence (%)	Optimal Modality	Pathognomonic Signs
Postoperative Pancreatic Fistula (POPF)	13–41	CECT Portal Venous/Delayed Phase	Peri-anastomotic fluid collection with elevated drain amylase
Delayed Gastric Emptying (DGE)	15–30	Clinical + CECT Portal Venous Phase	Persistent gastric distension with normal anastomoses
Biliary Leakage	5–8	MRI with HBA, Hepatobiliary Delayed Phase	HBA extravasation into collection on MRI
Fluid Collection/Abscesses	Up to 30	CECT/MRI	Hypoattenuating peri-anastomotic collections with or without gas collection within
Postoperative Hemorrhage	2–8	CECT Arterial Phase	Contrast extravasation; sentinel clot sign
Pancreatitis	Variable	CECT/MRI	Pancreatic enlargement; fat stranding; decreased apparent diffusion coefficient values (on MRI)
Anastomotic Strictures	Low	Contrast-Enhanced MRCP	Tapered biliary narrowing at anastomosis
Hepatic Infarction	Rare	CECT	Wedge-shaped hypoenhancing hepatic area
Splenic Infarction	Rare	CECT	Wedge-shaped hypoenhancing splenic area

## Data Availability

Data available on demand to the corresponding author.
